# Sudden death in young South European population: a cross-sectional study of postmortem cases

**DOI:** 10.1038/s41598-023-47502-0

**Published:** 2023-12-20

**Authors:** Mafalda Carrington, Rosa Henriques de Gouveia, Rogério Teixeira, Francisco Corte-Real, Lino Gonçalves, Rui Providência

**Affiliations:** 1 Department of Cardiology, Centro Hospitalar Vila Nova de Gaia/Espinho, Vila Nova de Gaia, Portugal; 2 Forensic Pathology Department, Delegação do Centro, Instituto Nacional de Medicina Legal e Ciências Forenses, Coimbra, Portugal; 3 Pathology and Histology, Faculty of Life Sciences, University of Madeira, Funchal, Madeira, Portugal; 4 LANA – Laboratory of Clinical and Anatomical Pathology, Funchal, Madeira, Portugal; 5 Medical Faculty, Coimbra University, Coimbra, Portugal; 6 Cardiology Department of Centro Hospitalar, Universitário de Coimbra, Coimbra, Portugal; 7 St Bartholomew’s Hospital, Barts Heart Centre, Barts Health NHS Trust, London, UK; 8 Institute of Health Informatics Research, University College of London, London, UK

**Keywords:** Arrhythmias, Cardiomyopathies, Acute coronary syndromes, Cardiac hypertrophy

## Abstract

To describe the annual incidence and the leading causes of sudden non-cardiac and cardiac death (SCD) in children and young adult Portuguese population. We retrospectively reviewed autopsy of sudden unexpected deaths reports from the Portuguese National Institute of Legal Medicine and Forensic Sciences’ database, between 2012 and 2016, for the central region of Portugal, Azores and Madeira (ages 1–40: 26% of the total population). During a 5-year period, 159 SD were identified, corresponding to an annual incidence of 2,4 (95%confidence interval, 1,5–3,6) per 100.000 people-years. Victims had a mean age of 32 ± 7 years-old, and 72,3% were male. There were 70,4% cardiac, 16,4% respiratory and 7,5% neurologic causes of SD. The most frequent cardiac anatomopathological diagnosis was atherosclerotic coronary artery disease (CAD) (33,0%). There were 15,2% victims with left ventricular hypertrophy, with a diagnosis of hypertrophic cardiomyopathy only possible in 2,7%. The prevalence of cardiac pathological findings of uncertain significance was 30,4%. In conclusion, the annual incidence of SD was low. Atherosclerotic CAD was diagnosed in 33,0% victims, suggesting the need to intensify primary prevention measures in the young. The high prevalence of pathological findings of uncertain significance emphasizes the importance of molecular autopsy and screening of first-degree relatives.

## Introduction

Sudden death (SD) in young individuals is relatively rare, with an estimated incidence of 4.6 per 100,000 person-years^1^. Yet, it remains an important health problem that frequently attracts *media* attention, as it affects previously healthy individuals, thus raising concerns about screening programs and carrying significant social and psychological consequences to the relatives of the victims.

The real prevalence of the different causes and main triggers of SD in the young is unknown in Portugal and uncertain in the world. In general, we know that sudden cardiac death (SCD) (Table 1) is more frequent than SD of noncardiac origin^2,3^. Furthermore, cardiac causes differ in young (< 40 years-old) *versus* older individuals, with a predominance of channelopathies, cardiomyopathies and myocarditis in the young^4,5^. In two European studies, sudden arrhythmic death syndrome (SADS) was the most prevalent cause of death in athletes and in the general population, followed by coronary artery anomalies and ischemic heart disease^3,6^.

**Table 1:** Definitions.

Natural death	A death caused solely by disease or natural processes, as opposed to violent death
Violent death	Death resulting from an homicide, suicide, accident and/or with a cause identifiable on toxicological analyses
Sudden death (SD)	A death that occurs within 1 h of onset of symptoms in witnessed cases, and within 24 h of last being seen alive when it is unwitnessed^4,5,13^
Sudden cardiac death (SCD)	In autopsied cases, SCD is a natural unexpected death of unknown or cardiac cause, as well as a death presumed to be of cardiac cause and that occurs within 1 h of onset of symptoms in witnessed cases, and within 24 h of last being seen alive when it is unwitnessed^4,5,13^
Molecular autopsy	The use of molecular techniques of genetic sequencing to try to determine the cause of death in unexplained cases
Sudden Arrhythmic Death Syndrome (SADS)	Unexplained sudden death occurring in an individual older than 1 year with negative pathological and toxicological assessment
Left Ventricular Hypertrophy (LVH)	Increased heart weight compared to normal predicted values^53^
LVH with evolution towards dilation	Increased heart weight compared to normal predicted values with cavity dilation^53^
LV dilation	Dilated left ventricular cavity with normal or reduced wall thickness
Ischemic heart disease	Cardiac dilatation due to scaring and remodelling of the myocardium, in an atherosclerotic ischemic context
Interstitial myocardial fibrosis	Increased deposition of connective tissue in the interstitium without myocytes loss^53^
Replacement-type myocardial fibrosis	Corresponds to myocardial scar. Increased deposition of connective tissue following extensive myocytes loss ^53^

Regarding cardiac arrest or SD of noncardiac origin, there is also a wide range of reported prevalence, ranging from 10 to 45%^7^ of the cases of SD in the general adult population. Specific studies of SD of noncardiac origin in young populations are scarce. Respiratory diseases seem to be the second more prevalent cause, followed by cerebrovascular causes^8,9^.

It is not infrequent that the definite SD etiology is only possible at *postmortem* examination. We believe that a representative study of the most common causes and triggers for SD of young individuals undergoing necropsy will help: (i) understand the natural history of the underlying diseases, and (ii) (re)define effective prevention measures and strategies for early rescue in the community and at emergency medical services level^10,11^.

We aimed to: (i) define the annual incidence of SD in the young Portuguese population (1 to 40 years-old); (ii) describe the leading causes of sudden cardiac and non-cardiac death, with a sub-analysis for different age groups; and (iii) identify sudden cardiac death triggers or risk factors.

## Material and methods

### Case selection

In a cross-sectional study, we retrospectively reviewed cases from the database of the Portuguese Institute of Legal Medicine and Forensic Sciences (INMLCF, I.P.) of individuals who underwent an autopsy between January 2012 and December 2016 (5-year period), at the Central Region Branch, comprising 7 districts of mainland Portugal plus Madeira and Azores. Medico-legal autopsies were conducted at the respective district Medical-Legal offices, and samples were subsequently sent to the Central Branch Headquarters in Coimbra for anatomopathological analysis, performed by one of the authors of this article (RHG), who specializes in cardiovascular pathology. This approach was taken to minimize potential bias in the analysis. Additionally, data collection and synthesis for the study were conducted jointly by a Cardiologist (MC) and the aforementioned Anatomo-Pathologist (RHG). Furthermore, the toxicological analysis has been standardized, with all procedures being carried out at the headquarters' laboratory. In Portugal, all victims of SD are supposed to undergo post-mortem examination.

**Figure 1:**
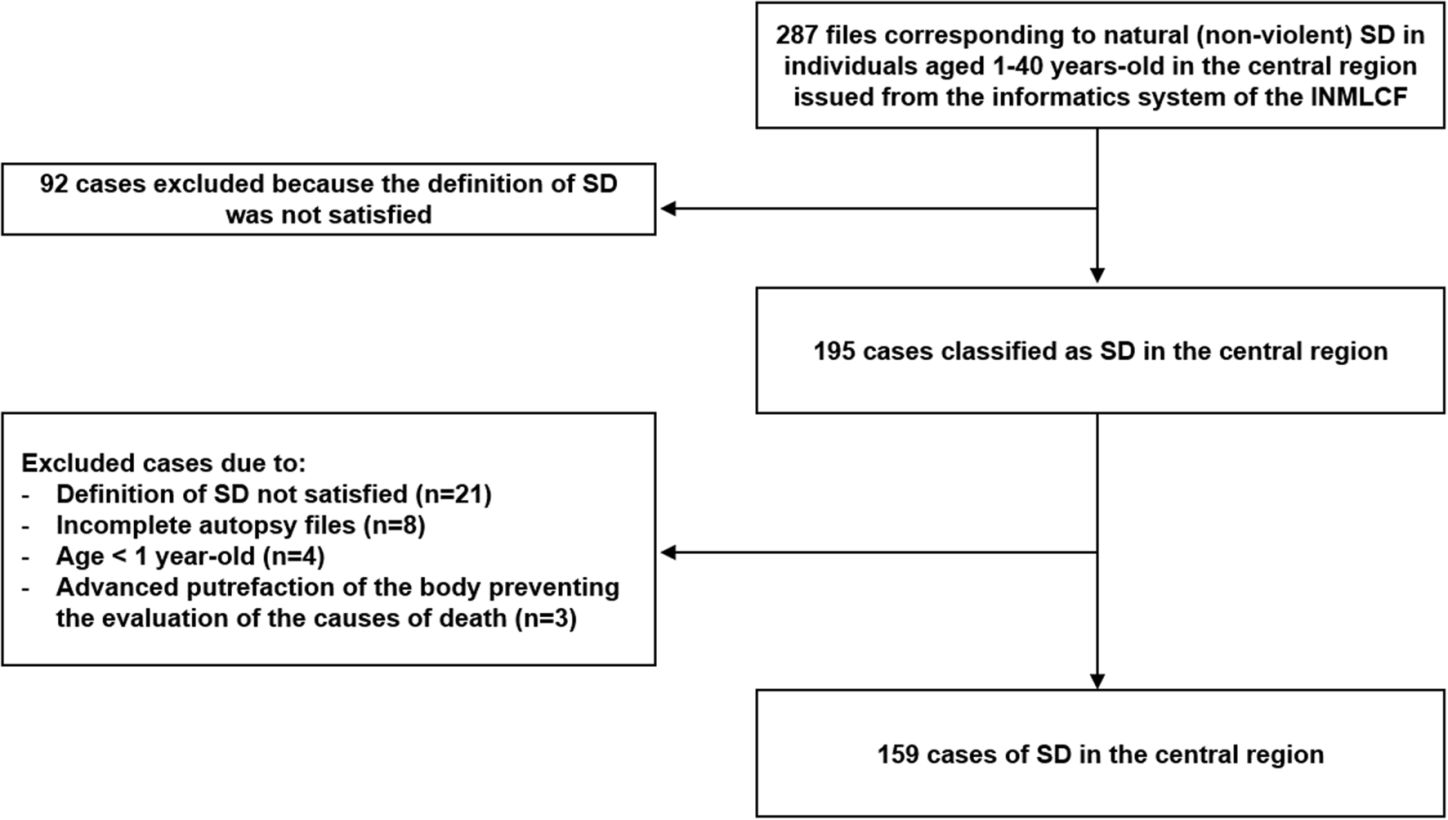
Flow chart of study identification of cases and exclusion criteria.

This study included young individuals who were classified by the physician who performed the autopsy to have had a sudden and unexpected death between the ages of 1 to 40. Based on the 2011 Census of the National Institute of Statistics of Portugal, among a total Portuguese population of 10.562.178 inhabitants, 5.034.869 individuals were aged 1 to 40 (47,7%), and, of these, 1.309.963 (26,0%) lived in the central region (n = 1.032.150, 20,5%), Madeira (n = 139.530, 2,8%) and Azores (n = 138.283, 2,7%). Sub-analysis of the leading causes of sudden cardiac and non-cardiac death were performed for different age groups: children and teenagers (≤ 18 years-old), early adulthood (19–34 years-old) and early middle-aged adults (35 to 40 years-old)^12^. A query with the words “1–40 yeard-old”, “natural death” and “sudden death” (Table 1) was performed to find the files corresponding to SD at the informatics system of INMLCF. We excluded victims in whom previous definition of SD was not satisfied (Table 1)^4,5,13^, individuals who had a violent death or a natural death—neither sudden nor unexpected, as well as cases in which the cause of death could not be determined due to advanced putrefaction of the body or due to incomplete autopsy file (Fig. 1).

This study was approved by the INMLCF’s Ethics Committee (INMLCF-30/05/2018) that waived informed consent from the victim’s families. All research was performed in accordance with Ethics Committee and Information Access Responsible regulations.

### Variables

We analyzed the autopsy files of the included individuals and selected demographic (age and gender), clinical (medical history, addiction habits, risk factors), circumstantial (place and manner of death, witnesses, symptoms one hour before death and resuscitation maneuvers) necropsic (external and internal habitus), anatomopathological (macroscopic and histological organ evaluation) and toxicological variables. Anatomopathological evaluation was available in all expect three cases and toxicological analysis was possible in all cases. Genetic investigation was known in two cases. Available clinical and circumstantial variables were collected based on the textual description present in the autopsy report. Prior medication information was obtained based on the description provided in the autopsy and toxicology report. The final cause of death was ascertained based on the data present in the autopsy report (after toxicological and the anatomopathological diagnosis), in the light of the Guidelines for autopsy investigation of sudden cardiac death (2017)^5,14^. Finally, deaths in which both autopsy and toxicology investigations were inconclusive, non-cardiac etiologies were excluded, and the heart had non-diagnostic pathological findings of uncertain significance at gross and histological examination, ^15^ were classified as possible SADS as the final cause of death^4^.

In the anatomopathological reports of autopsies, coronary atherosclerotic lesions are categorized based on the Committee on Vascular Lesions of the Council on Atherosclerosis from the American Heart Association (AHA) classification^16^. Therefore and according to the Fourth Universal Definition of Myocardial Infarction^17^, the histopathological diagnosis of type VI AHA complicated lesions with a surface defect, hematoma hemorrhage and/or thrombotic deposit led to the diagnosis of type 1 MI as the final cause of death. According to a document endorsed by the Association for European Cardiovascular Pathology^18^, coronary artery disease is considered as a highly probable cause of death when a stable atherosclerotic plaque with luminal stenosis > 75% is found with or without healed myocardial infarction; if an acute coronary occlusion is present (with or without myocardial infarction), this is considered as a certain cause. Therefore, the remaining individuals were classified has having presumed type 2 MI if they had no other clear cardiac or non-cardiac cause of SD and significant atherosclerotic lesions (AHA type IV and V), with an estimated luminal narrowing from 50 to 75% or more than 75% were present.

### Statistical analysis

We performed statistical analysis using Stata 13.0 software. Incidence rates were calculated using the 2011 Portugal Census age group population as a denominator. Confidence intervals (CIs) for incidence rates were calculated using the Poisson distribution. Categorical variables were described as numbers of cases and percentages, and continuous variables as means ± standard deviation (StDev). We used χ^2^ or Fisher exact test for categorical variables (as appropriate) and Student t test for continuous, to determine whether the presence of demographic, clinical and management features differed between individuals with the different causes of SD. To express the strength of these relations, we obtained the odds ratios (OR) with 95% confidence intervals through univariate logistic regression. A *p*-value < 0.05 was regarded as significant and two-tailed tests were applied. We obtained sample data from the INMLCF, I.P. database, consisting of individuals who underwent autopsies over a 5-year period at the Central Region Branch. This dataset represents a cross-section of our country's population and, as such, we deemed it to be comprehensive and readily available, covering the entire population of interest. Consequently, we determined that performing a formal sample size calculation was unnecessary.

## Results

**Figure 2:**
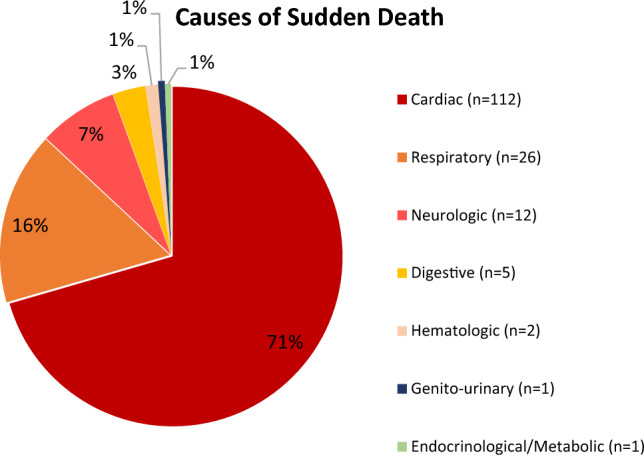
Causes of Sudden Death.

During a 5-year period, 159 SD were identified in individuals aged 1 to 40 years-old, corresponding to an annual incidence of 2,4 (95%CI 1,5–3,6) per 100.000 people-years. SD victims had a mean age of 32 ± 7 years-old, the majority being of male gender (72,3%, n = 115). There were 112 (70,4%) cases of SD of cardiac origin. The remaining causes are depicted in Fig. 2. Non-cardiac causes of sudden death are detailed in Table 2.

**Table 2:** Attributed causes of non-cardiac sudden death.

Non-cardiac causes of sudden death	N = 47, n (%)
**Respiratory**	26 (55,3)
* Lower respiratory tract infections*	
Acute bronchopneumonia	7 (14,9)
Acute lobar pneumonia	7 (14,9)
Acute purulent bronchitis	2 (4,2)
* Upper respiratory tract infections*	
Acute laryngitis	1 (2,1)
Acute severe amygdalitis with prominent lymphoid follicular hyperplasia	1 (2,1)
Acute alveolar hemorrhage	3 (6,4)
Acute diffuse alveolar disease	3 (6,4)
Acute pulmonary edema	
in the context of Still’s Disease	1 (2,1)
in the context of Sickle Cell Anemia crisis	1 (2,1)
**Neurologic**	12 (25,5)
Hemorrhagic stroke	4 (8,5)
Generalized tonic–clonic seizure	4 (8,5)
Subarachnoid hemorrhage	2 (4,2)
Acute meningitis	1 (2,1)
Brain ventriculitis and acute cervical medullary hemorrhage	1 (2,1)
**Digestive**	5 (10,6)
Acute peritonitis	
Secondary to gastric ulcer perforation	2 (4,2)
Secondary to intestinal perforation	1 (2,1)
Gastric hemorrhage	1 (2,1)
Granulomatous necrotizing hepatitis	1 (2,1)
**Hematologic**	2 (4,2)
Intravascular disseminated coagulation secondary to severe sepsis to Streptococcus suis	1 (2,1)
Lymphoproliferative disease	1 (2,1)
**Genito-urinary**	1 (2,1)
Ulcerated infected uterine cervix carcinoma	1 (2,1)
**Endocrinological/Metabolic**	1 (2,1)
Severe hypoglycemia in the context of insulin-treated DM	1 (2,1)

DM, Diabetes Mellitus.

### Attributed causes of sudden cardiac death

Anatomopathological diagnoses obtained in SCD victims are depicted in Table 3, with a descending order of frequency. The most frequent cardiac diagnosis was atherosclerotic coronary artery disease (CAD) (n = 37, 33,0%), which included cases of documented type 1 AMI (AHA type VI lesion) (n = 16, 43,2%), presumed type 2 AMI (AHA type IV or V lesions) with an estimated luminal narrowing from 50 to 75% (n = 6, 16,2%) or more than 75% (n = 13, 35,1%), one case in which coronary arteries were not sent for anatomopathological analysis but histological myocardial infarction was detected, and another in which no histology was requested although macroscopic examination detected myocardial wall infarction and rupture with associated hemopericardium. A minority of victims with atherosclerotic CAD had histological evidence of myocardial infarction (n = 12, 32,4%), three of which corresponded to presumed type 2 AMI. AMI as a final cause of SCD could then be confirmed in twenty-one (18,8%) individuals who had evidence of myocardial ischemic lesions and/or type 1 AMI. There was no evidence of interstitial or replacement-type myocardial fibrosis in the remaining cases of suspected type 2 AMI (n = 16, 14,3%).

**Table 3:** Attributed causes of sudden cardiac death.

Main anatomopathological diagnosis	N = 112, n (%)	Mean age ± SD (years-old)	Gender predominance
**Atherosclerotic coronary artery disease**	37 (33,0)	34 ± 4 (*p* < 0,047)	Male (83,8%; *p* = 0,032)
Type VI complicated lesion (AHA classification) *	16 (14,3)		
LVH**	17 (15,2)	33 ± 8	NS
Evolution towards dilation	8 (7,1)		
Associated with interstitial fibrosis	4 (3,6)		
With extensive area of replacement-type fibrosis	2 (1,8)		
Associated myxomatous mitral valve disease	1 (0,9)		
Hypertrophic CM***	3 (2,7)	19 ± 9,5 (*p* = 0,001)	NS
**Acute pulmonary embolism**	14 (12,5)	33 ± 6	Women (78,6%; *p* < 0,001)
With documented deep venous thrombosis	9 (8,0)		
**Dilated LV**	10 (8,9)		
Probable etiology:			
Post-partum	2 (1,8)	31 ± 8	NS
Ethanolic	2 (1,8)		
Ischemic	1 (0,9)		
Post-myocarditis	1 (0,9)		
**Valvular Heart disease**	7 (6,2)	36 ± 4	NS
Myxomatous mitral valve disease	4 (3,6)		
Severe aortic stenosis	2 (1,8)		
Degenerative mitral valve disease	1 (0,9)		
**Acute myocarditis**	5 (4,5)	20 ± 11 (*p* = 0,002)	NS
**Acute pulmonary edema/acute heart failure**	5 (4,5)	34 ± 5	NS
**Ascending aorta dissection and pericardial tamponade**	5 (4,5)	30 ± 8	NS
**Congenital Heart Disease**	5 (4,5)	33 ± 4	NS
Corrected	3 (2,7)		
**Left ventricular fibrosis**	2 (1,8)	38 ± 4	NS
Mild and multifocal interstitial fibrosis	1 (0,9)		
Replacement-type fibrosis	1 (0,9)		
**Arrhythmogenic Right Ventricle CM**	1 (0,9)	30	NS
**Acute left main coronary artery dissection**	1 (0,9)	24	NS

*Corresponds to clinical type 1 myocardial infarction, ** Not meeting criteria for Hypertrophic CM, ***Two cases with anatomopathological and genetic data, one case with a previously known diagnosis and whose heart was not sent for anatomopathological analysis.

AHA, American Heart Association; CM, Cardiomyopathy, NS, non-significant.

There were three cases of HCM, including one victim with 30 years-old who had a previously known diagnosis and whose heart was not sent for anatomopathological analysis, and another two children, with genetic evaluation and autopsy macro and microscopic typical pattern (supplementary material 1 and cases 55–57 from supplementary material 2).

Left ventricular (LV) dilation and associated interstitial fibrosis was present in two cases, one of which was a victim who had previous coronary artery bypass grafting (CABG). No significant bypass stenosis at gross and histological examination was observed in that patient and a final diagnosis of ischemic heart disease with an anterior myocardial scar, complicated with acute pulmonary edema was made (supplementary material 1). A presumptive etiology was suggested for five other cases of LV dilation: two SD occurred three- and four-months following delivery, probably in the context of an arrhythmia secondary to post-partum cardiomyopathy in 37- and 17-years-old females, respectively. The older victim also had an history of alcohol abuse and significant liver disease (case 74 from supplementary material 2). Two other cases also had a history of alcohol abuse and possibly corresponded to ethanolic cardiomyopathy (cases 75 and 81); the fifth case corresponded to a male aged 18, without previous relevant medical history, where, despite the absence of interstitial fibrosis or histological evidence of myocarditis on the dilated ventricle, additional features on anatomopathological examination raised the hypothesis of subacute myocarditis due to a viral infection or auto-immune disease (case 72).

Among four individuals with myxomatous mitral valve disease, two had mitral valve prolapse, three had associated left ventricular hypertrophy (LVH) and one also had peri-valvular basal ventricular interstitial fibrosis.

Among the five cases (4,5%) of acute myocarditis identified, there was a 15-years-old boy with associated mild left ventricular dilation and interstitial fibrosis and a 23-years-old woman with simultaneous active peri-coronary inflammation, as well as small myocardial scars suggesting previous myocarditis episodes.

All victims with a final diagnosis of acute pulmonary edema/acute heart failure (n = 5, 4,5%) had no underlying cardiac structural abnormalities, and evidence of edema involving more than 50% of the lungs was present, frequently accompanied by generalized vascular congestion.

Among victims with ascending aorta dissection and pericardial tamponade (n = 5, 4,5%), a dissection arose in a pre-coarctation aneurysm, in which chronic peri-aortic inflammation was observed (case 99). Two other individuals had simultaneous non-significant CAD and one had significant 1-vessel CAD (50–75% stenosis).

Acute left main coronary artery dissection originating from medial tunica mucoid degeneration [extra-cellular mucoid matrix accumulation (MEMA)]^19^ of the ascending aorta was observed in one male subject. Further examination also revealed mitral valve myxomatous changes, suggesting a hereditary Marfan Syndrome (case 112).

### Causes of sudden death by age strata

**Figure 3:**
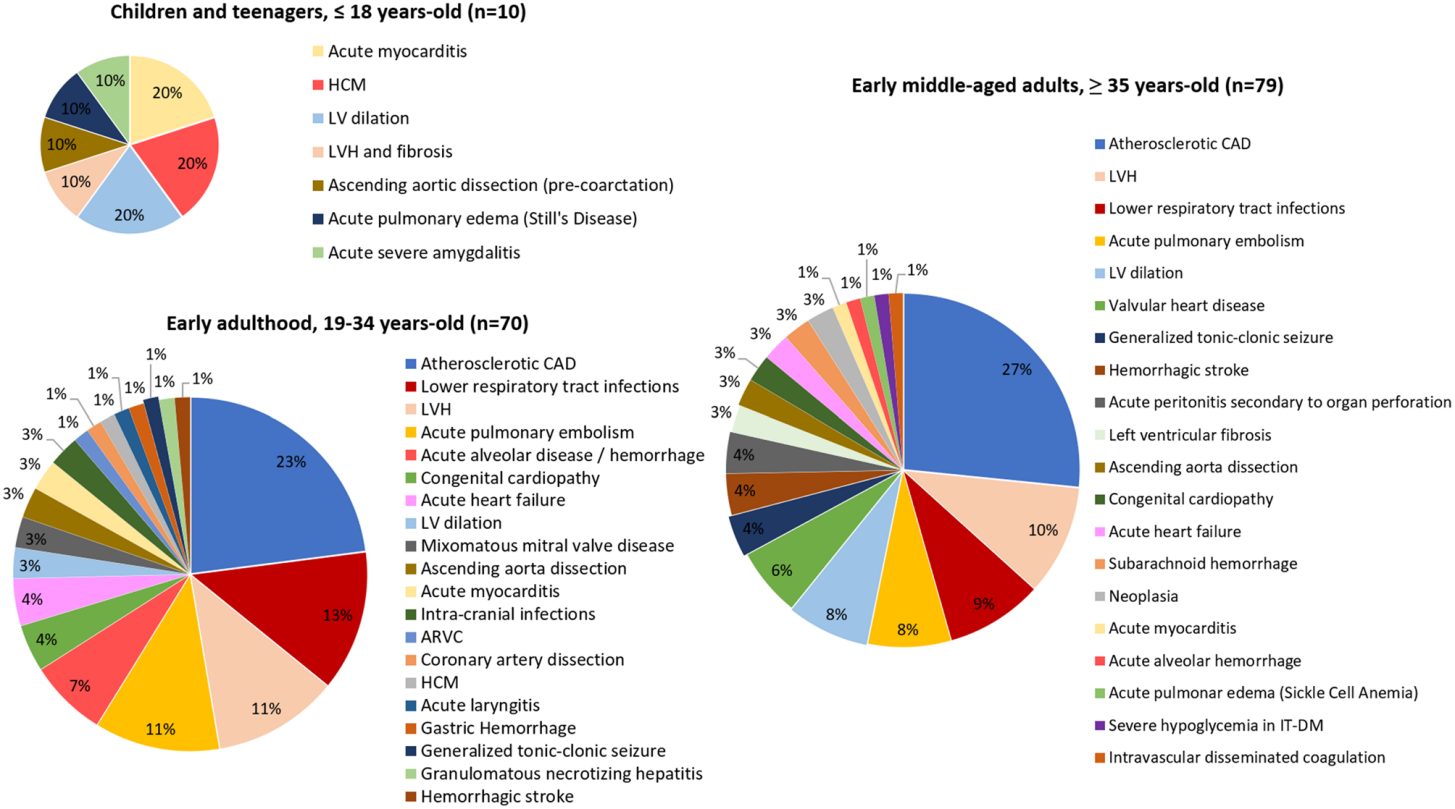
Attributed causes of sudden death by age strata.

In order to analyze autopsy final causes of SD after anatomopathological diagnosis across age strata, we separated children and teenagers (≤ 18 years-old, n = 10, 6,3%), from early adulthood (19–34 years-old, n = 70, 44,0%) and from early middle-aged adults (35 to 40 years-old, n = 79, 49,7%) (Fig. 3)^12^. There was a growing annual incidence of SD among age strata: 0,4 (95%CI 0,3–0,5), 2,5 (95%CI 1,6–3,7) and 6,3 (95%CI 4,8–8,1) per 100.000 people-years, respectively.

Among victims aged ≤ 18 years-old, the most common cause of SD was acute myocarditis (n = 2), HCM (n = 2) and dilated LV (n = 2), one of which corresponded to the case of subacute myocarditis with associated hepatitis and BALT hyperplasia, as discussed in the previous section. This younger group included a case of a 11-years-old girl who had LVH and interstitial fibrosis, including an extensive area of myocardial scar, also with associated BALT hyperplasia, probably secondary to previous myocarditis (case 44). Even though myocarditis may have been a direct or indirect cause of SD in 40,0% of these individuals (n = 4; cases 44, 72, 90, 91 from supplementary material 2), the prevalence of confirmed acute myocarditis was 21 times higher in children and teenagers (30,0%) than in middle-aged adults (2,0%) (OR 20,8; 95%CI 3,55–122,56, *p* = 0,001).

In adults, the most common diagnosis was atherosclerotic CAD, with a non-significant tendency to be more frequent in early middle-aged adults (27% vs. 20%, OR 1,45; CI 0,69–3,04, *p* = 0,327) comparing to younger individuals. A similar tendency was observed in AMI as a final cause of SD, which was present in 16,5% (n = 13) of early middle-aged adults and 11,4% (n = 8) in early adulthood (*p* = 0,381). All expect 3 of these victims with AMI were 30 years-old or older. The subsequent three most common findings were LVH, lower respiratory tract infections and acute pulmonary embolism. There were no statistically significant differences when comparing diagnoses between early adulthood *versus* early middle-aged adults.

### Toxicological analysis

In the toxicological analysis, there were two individuals with the final diagnosis of type 1 AMI and one with presumed type 2 AMI who had positive non-toxic cannabinoids plasma levels and none had positive cocaine levels or other drugs. In addition, there were fifty (31,4%) victims who had positive non-toxic levels of medications, eight (5,0%) with non-toxic alcohol and four (2,5%) with non-toxic cannabinoids levels.

### Clinical characteristics of sudden cardiac death victims

Addiction habits were reported in 31 (27,7%) cases and detected in 22 (71,0%) of those individuals, including 13 with alcohol abuse, eight with intravenous or non-intravenous drug abuse and/or five who smoked. There were 39 (34,8%) individuals who had prior medication, including 27 (69,2%) who were on psychotropics. In addition, there were eight (7,1%) victims who had anticonvulsant medication or a prior diagnosis of epilepsy. Among them, the cause of death was potentially associated with recurrent syncope in two cases: Arrhythmogenic Right Ventricle Cardiomyopathy (ARVC) and severe aortic stenosis.

Victims with prior cardiovascular history and risk factors, and their attributed causes of SCD are depicted in supplementary material 1. There were 16 (14,2%) who had at least one cardiovascular risk factor reported and seven (6,2%) had two or more. Among these, eight had autopsy anatomopathological diagnosis of atherosclerotic CAD, seven were diagnosed with LVH and one died from acute ascending aorta dissection. Furthermore, one victim aged 36 years-old had previous CABG surgery but no history of known risk factors. A 32-year-old man with non-toxic cannabinoid plasmatic levels and a previous diagnosis of Wolff-Parkinson-White Syndrome, had AHA type 4 and 5 lesions in the anterior descending artery causing a stenosis superior to 75% and a diagnosis of type 2 AMI was presumed. Finally, there were two individuals who had a previous history of unclassified arrhythmia and received the diagnosis of myxomatous mitral valve disease and of LVH, interstitial and replacement-type myocardial fibrosis, respectively.

### Circumstances of collapse/death in sudden cardiac death victims

**Figure 4:**
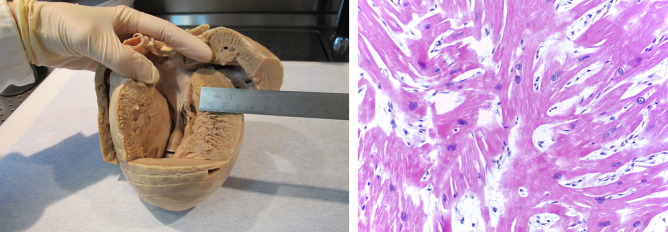
Representative macroscopic and microscopic figure of a HCM case. Case 57: 14 years-old boy, HCM, heart = 740g weight, left ventricle (LV) = 25–30 mm thick, IV Septum = 35 mm thick, right ventricle (RV) = 6 mm. Macroscopic view of the heart (left) and microscopic section of myocardial disarray in HCM (HE × 400) (right). Source: *INMLCF.*

Among individuals with a cardiac cause of SD, the place and circumstances of collapse are described in Table 4. Among victims who died during the night, there were two males and one female aged 31–40 years-old, all with LVH (cases 46, 48, 53), a 31-years-old female with mitral valve prolapse (case 82) and a 36 years-old man with atherosclerotic CAD (AHA type IV lesion, case 20), all without evidence of ischemic lesions, interstitial or replacement-type myocardial fibrosis. Moreover, six (15,0%) SD occurred during or immediately after exertion: two individuals with a final diagnosis of type 1 AMI collapsed during physical effort at work (case 17) and after housework (case 27), respectively, one boy with HCM collapsed while running away from a dog (case 55), another with HCM died during the physical education class (case 57, Fig. 4), one man with myxomatous mitral valve disease collapsed while playing football (case 84) and another man who collapsed immediately after playing football received the anatomopathological diagnosis of LVH and associated mild interstitial fibrosis, but no myocardial scar (case 38).

**Table 4:** Circumstances of collapse/death in sudden cardiac death victims.

Reported circumstances of collapse/death	N = 112, n (%)
**Place of collapse/death**	62 (55,4)
Home	35 (56,4)
Hospital/other health institution	11 (17,7)
Work	6 (9,7)
Public place	6 (9,7)
Entertainment place	4 (6,4)
**Circumstances of collapse**	40 (35,7)
Unknown—found death/after loss of consciousness	19 (47,5)
At rest	15 (37,5)
During sleep	7 (17,5)
While in/immediately after exertion	6 (15,0)
**Witness**	36 (32,1)
Yes, of collapse	24 (66,7)
Yes, of the good health status 24 h before the event	12 (33,3)
**Symptoms 1 h before death**	51 (45,5)
**Yes**	19 (37,2)
Chest pain	6 (11,7)
Syncope/pre-syncope	4 (7,8)
Indisposition	3 (5,8)
Vomiting	2 (3,9)
Unknown	2 (3,9)
Palpitation	1 (2,0)
Dyspnea	1 (2,0)
**No**	6 (11,8)
**Unknown in individuals who were found collapsed/death**	26 (51,0)
**Cardiopulmonary resuscitation**	55 (49,1)
**Yes**	42 (76,4)
Pre-hospital	24 (43,6)
In-hospital	7 (12,7)
Unknown setting	11 (20,0)
**No**	13 (23,6)

Symptomatic status was considered unknown in 26 victims who were found dead or who died during sleep. Cardiopulmonary resuscitation (CPR) was performed in 42 (76,4%), the majority in pre-hospital setting including three cases who also received CPR from relatives and colleagues.

## Discussion

We analyzed 159 autopsy reports and causes of SD in a cohort of young (1–40 years-old) Portuguese SD victims who underwent autopsy in the central region of the country during a 5-year period.

In line with previous reports, the annual incidence of SD in victims aged 1–40 years was 2,4 per 100.000 people-years^3, 20^, the majority of SD were of cardiac origin and neurological and respiratory diseases were the most frequently reported noncardiac causes of SD^2, 3, 21, 22^. Similarly to our findings, a Dutch nationwide study identified 33,5% prevalence of AMI as a cause of SCD^2, 23^. In contrast, a Danish nationwide study, including a slightly younger population (median age 29^22–33^), reported a prevalence of 12,7% of ischemic heart disease and found no cause of death in 29% of autopsied SD, suggesting a primary arrhythmogenic cause of death^3^. In our cohort, it is important to differentiate atherosclerotic CAD from AMI as a cause of SCD: although we presumed type 2 MI in cases with significant atherosclerotic lesions, with an estimated luminal narrowing from ≥ 50% and no other clear cardiac or non-cardiac cause of SD, histological AMI could not be confirmed in sixteen victims (14,3%). Although we cannot exclude that a transient coronary spasm on a stenotic coronary artery could lead to lethal arrhythmia without causing histological AMI, this is a clinical diagnosis of which we cannot be certain of by analyzing anatomopathological data of these cases. Therefore, we propose that these individuals may have CAD as an innocent bystander (not as the definite cause of death) and consequently the final cause of death is considered uncertain after autopsy. Indeed, the prevalence of coronary atherosclerosis is high even in the young ^24^: it was estimated to be 60% in heart transplant donors aged 30–39 years, 37% in donors aged 20–29 years^25^ and even in sports-related sudden cardiac arrests in individuals aged 18–35 years, CAD was also the most frequent etiology in 26% of the cases^11^.

Similarly, nearly a third of cases (30,4%) had pathological findings of uncertain significance: LVH, LV dilation without evidence of myocardial scar (n = 15 and n = 9, respectively), myxomatous (n = 4)^26^ or degenerative (n = 1) mitral valve disease, acute pulmonary edema or acute heart failure (n = 5) with coexisting LV interstitial fibrosis^15, 27^. These pathological findings could not categorically be classified as culprit, yet they may represent innocent bystanders, and thus underlie possible cases of SADS. In a younger population (aged 24 ± 10 years) of SCD victims, with similar male predominance (72%), Bagnall et al. found that unexplained SCD accounted for 40% of the cases^6, 28^. A clinically relevant cardiac gene mutation was identified in 27% of these victims, and 29% had structural cardiac changes such as LVH, dilation and/or interstitial fibrosis^28^.

In our study, among seven victims who died during sleep, five had non-diagnostic pathological findings and could have had a SCD due to long QT type 3 and Brugada Syndrome, as these patients with SCN5A gene mutations are typically young adult males who die during the night^29^. Similarly, the young man who collapsed immediately after playing football and was diagnosed with LVH could have had a SCD secondary to long QT type 1 (gene KCNQ1) or catecholaminergic polymorphic ventricular tachycardia (CPVT) (gene *RyR2*)^29^. Although there are some financial and bureaucratic constraints in our country, molecular autopsies in these cases could have led to the identification of pathogenic genes and thus have a significant impact in victims’ families^4^. In addition, genetic testing should be integrated with circumstances of death and family history as part of the multidisciplinary management of sudden cardiac death. Among SCD cases, *Lahrouchi* and colleagues identified a clinically actionable pathogenic or likely pathogenic variant in only 13% of the victims who underwent molecular autopsies^30^. However, combining clinical evaluation to genetic testing in surviving relatives of these victims increased diagnostic yield from 26 to 39% in family members^30^. In another article where the authors aimed to identify the spectrum of genes implicated in autopsy-inconclusive SCD, 22,0% disease-causing variants were identified. This study demonstrates that concealed cardiomyopathy should also be suspected in these cases, as cardiomyopathy-associated genes corresponded to 70% and were overrepresented in cases with sub-diagnostic structural findings at autopsy^31^. Finally, screening relatives within these families to identify cases of inherited cardiomyopathies or channelopathies can be of utmost importance for their prognosis and may result in the reclassification of the cause of death for deceased probands with previously uncertain diagnoses.

In our study, nearly one tenth of the SCD victims had anticonvulsant medication or a prior diagnosis of epilepsy as possibly occurring in sudden unexpected death in epilepsy (SUDEP). Differential diagnosis with arrhythmic events and syncope occurring in the context of ARVC or severe aortic stenosis must be done, since they may have been misdiagnosed as seizures. A similar (7%) prevalence of epilepsy was described in arrhythmic SCD victims, all of them having a long-term history of syncope and seizures before death^30^. Genetic testing identified a likely cause of death in 24% of these cases^30^. In addition, in our series, there were four neurological cases of SD in whom a generalized tonic–clonic seizure was described in the autopsy report and who may also be classified as SUDEP.

The second cardiac anatomopathological diagnosis was LVH (15,2%), which was associated with myocardial scar in only 2 cases and, as mentioned, could represent a bystander in victims of potential SADS. It is important to emphasize that despite presenting LVH, these individuals did not exhibit classic myocyte disarray, the historically histological hallmark of HCM, which was detected as final cause of SCD in only 2 cases. Other investigators reported prevalence ranging from 13 to 16% for idiopathic LVH as a cause of SCD in young athletes^6, 32^. However, we preferred not to use the term idiopathic, as in most of our cases we could not exclude other causes of LVH such as intensive physical training and hypertension, the latter being of particularly relevance in our Portuguese population which has high rates of uncontrolled hypertension and salt intake, even in the young^33, 34^. Interestingly, *Papadakis* et al. reported that in SCD cases where LVH or myocardial interstitial fibrosis was reported at postmortem, evaluation of family relatives identified a primary arrhythmogenic syndrome in half of the families^15^.

### Limitations

Some limitations to our study need to be emphasized. First, the retrospective nature of our data may be subject to bias. This may be particularly relevant in data presented in Table 4, where the number of missing cases is high in most of the variables, as well as in the classification of certain non-cardiac causes of SD such as pneumonia, peritonitis, sepsis and neoplasia (lymphoproliferative disease and cervix carcinoma) in which victims may have felt unwell before, although the symptoms’ description in the autopsy reports satisfy the criteria of SD. In addition, the toxicological analysis was all done at the Headquarters’ Laboratory; the anatomopathological study was uniformly done by the same Anatomo-Pathologist (with sub-specialization in cardiovascular pathology, RHG) at the Headquarters’ Laboratory and the data for the study was collected and synthetized by a Cardiologist (MC) and the aforementioned Anatomo-Pathologist (RHG). We analyzed autopsy reports performed by different physicians with Medico-Legal expertise and working in 9 different medico-legal offices in Portugal. This may have led to some interpersonal bias in classification, despite the general common approach guidelines. Finally, the incidence of SD may be slightly underestimated as we excluded cases in which the body presented advanced putrefaction, preventing the evaluation of the causes of death.

Epidemiological data on sudden cardiac death, with clarification of regional variations on etiology and causes, namely from low and middle-income countries are generally lacking. Furthermore, the available studies are usually small case series, with the uptake of necropsy studies being sometimes below the expected levels. Cultural, financial barriers and lack of experienced forensic professionals, may be some of the causes for the very low rate of autopsies in some areas^35^.

A recent systematic review identified high variability with uneven distribution of cardiac arrest and SCD registries around the globe^36^. Most seem to be located in North America and Western Europe. No currently active registries were identified in Africa, Russia, India, China or South America^36^. In addition, some conditions are not routinely investigated in some part of the World. For example, data on hereditary arrhythmic disorders of cardiomyopathies in Africa are sparse, with a few small case series on arrhythmogenic cardiomyopathy^37^, Brugada syndrome^38^ and long QT syndrome^39^. This means that, it is possible that some of these entities may be underdiagnosed in epidemiologic studies of SCD or necropsic studies in this continent. In conclusion, there is a crying need for better understanding of the ethnic and regional differences in the etiology of SCD (Supplementary Table 3), and more research is needed to fully understand the scope of the problem, namely in the African, South American and Asian continents^40^.

## Conclusions

During a 5-year period, the annual incidence of SD between ages of 1 to 40 was 2,4 (95%CI 1,5–3,6) per 100.000 people-years and 70,2% of these deaths were of cardiac origin. The most frequent cardiac anatomopathologic diagnosis was atherosclerotic CAD (33,0%) and AMI was present as the final cause of SCD in 18,8% of the cases. Although further studies are needed to establish the underlying significance of CAD with stenosis ≥ 50% in a third of SCD victims in Portugal, we suggest that reinforcing primary prevention measures in individuals younger than 40 and screening for familial hypercholesterolemia in surviving relatives may be important measures to implement. Finally, the prevalence of cardiac pathological findings of uncertain significance was 30,4%. These deaths could be related to primary arrhythmic syndromes, suggesting a role for molecular autopsy and implementation of screening of victims’ families.

### Supplementary Information

Supplementary Information 1.

Supplementary Information 2.

Supplementary Information 3.

## Data Availability

Data are available on request from the authors (please contact the first and corresponding author at mafaldacarrington@gmail.com) and in article Supplementary Material.
