# Supplementary material for: Sudden death in young South European population: a cross-sectional study of postmortem cases
Source: Sci Rep. 2023 Dec 20;13:22734. doi: 10.1038/s41598-023-47502-0 (PMC10733430; doi:10.1038/s41598-023-47502-0)
Supplement: Supplementary file 1 — Supplementary Information 1. [file 41598_2023_47502_MOESM1_ESM.pdf]

Supplementary material 2 – Victims with prior cardiovascular history and risk factors

Gender	Age	Prior cardiovascular history and risk factors	Attributed cause of SCD	Additional features
M	36	Dyslipidemia	Atherosclerotic CAD (type 2 MI)	2-vessel disease (50-75%), LV dilation
M	29	Smoking	LVH	Associated myxomatous mitral valve disease
M	39	Insulin-treated DM	LVH with evolution towards dilation	Diffuse glomerulosclerosis and focal lamellar sclerosis suggestive of diabetic and/or hypertensive nephropathy
F	34	Insulin-treated DM	Acute MI (type 2)	1-vessel disease (50-75%), myocardial fibrose and scar
M	37	Smoking	Acute MI (type 1)	3-vessel disease, LV dilation
M	36	Obesity	Acute MI (type 2)	1-vessel disease (75-90%), myocardial scar
M	23	Medicated arterial hypertension	LVH with evolution towards dilation	Biventricular hypertrophy, non-significant CAD; Adrenal cortical adenoma
F	40	Morbid obesity	LVH with evolution towards dilation	Non-significant CAD
M	40	DM	LVH	Patent *foramen ovale*
F	39	DM and obesity	LVH with interstitial fibrosis	Non-significant CAD
M	31	Arterial hypertension and dyslipidemia	LVH with evolution towards dilation	Non-significant CAD, acute pulmonary edema and generalized vascular congestion; Hypertensive glomerular arteriosclerosis
M	32	Arterial hypertension and dyslipidemia	DeBakey type I aortic dissection and pericardial tamponade	Ascending aorta sacular aneurysm, aortic atherosclerosis and *media* degeneration with acid mucopolysaccharide deposition; cardiopathy with biventricular hypertrophy; 1-vessel significant disease (50-75%)
M	36	Arterial hypertension and obesity	Atherosclerotic CAD (type 2 MI)	1-vessel disease (>75%), LVH
M	40	Smoking and obesity	Atherosclerotic CAD with AHA type 6 lesion (type 1 MI)	1-vessel disease
M	39	Arterial hypertension, smoking and obesity	Acute MI (type 1)	3-vessel disease, LVH
M	28	Arterial hypertension, smoking and obesity	Acute MI (type 1)	2-vessel disease, LVH and evolution towards dilation
M	30	Obstructive HCM, medicated and awaiting surgical septal myectomy	No anatomopathological evaluation requested	
**M**	14	Familial non-obstructive HCM, medicated with atenolol	Obstructive HCM	Heart weight: 740g, interventricular septum thickness of 35mm. Myocyte disarray. Two pathogenic MYBPC3 gene mutations: *antemortem* diagnosis of father-inherited mutation and *postmortem* diagnosis of mother-inherited mutation. Toxicology: atenolol in the therapeutic range.
M	32	Wolff-Parkinson-White Syndrome Cannabinoid consumption	Atherosclerotic CAD (type 2 MI)	LVH; persistent left superior vena cava draining into the coronary sinus
M	24	Unclassified arrhythmia	LVH with interstitial and replacement-type fibrosis	Extensive anterolateral scar with compensatory hypertrophy, RV dilation, small caliber left coronary arteries
M	36	Unclassified arrhythmia	Myxomatous mitral and tricuspid valve disease	LVH, RV and bi-atrial dilation; patent *foramen ovale*
M	32	Heart murmur, restriction to sports participation	Congenital Heart Disease	Pulmonary artery agenesia, interatrial communication, persistent left superior vena cava draining into aneurysmatic coronary sinus, left atrial and mitral valve hypoplasia
M	36	Previous CABG surgery	Ischemic heart disease	Complicated with acute pulmonary edema
M	27	Right heart valvuloplasty (3 months before SD)	No anatomopathological evaluation requested	
F	36	Surgical repair of stenotic pulmonary artery	Corrected Congenital Heart Disease	Bicuspid pulmonary valve and sub- and supra-valvular stenosis corrected with a pulmonary trunk patch; cardiopathy with biventricular hypertrophy, interstitial and replacement-type fibrosis, tricuspid annulus dilation.
M	31	Surgical repair of pulmonary valve atresia	Corrected Congenital Heart Disease	Pulmonary valve with dysplastic cuspids and a calcified patch in the RV outflow tract; cardiopathy with biventricular dilation, hypertrophy, interstitial and replacement-type fibrosis involving predominantly the RV

Legend: Orange lines correspond to prior cardiovascular risk factors, yellow lines to prior cardiovascular diseases and green lines to prior cardiovascular interventions.

CABG – coronary artery bypass grafting, CAD – coronary artery disease, DM – Diabetes Mellitus, HCM – Hypertrophic Cardiomyopathy, LVH – Left Ventricular Hypertrophy, MI – Myocardial Infarction, RV – right ventricle
