# Supplementary material for: Sudden death in young South European population: a cross-sectional study of postmortem cases
Source: Sci Rep. 2023 Dec 20;13:22734. doi: 10.1038/s41598-023-47502-0 (PMC10733430; doi:10.1038/s41598-023-47502-0)
Supplement: Supplementary file 2 — Supplementary Information 2. [file 41598_2023_47502_MOESM2_ESM.pdf]

Supplementary material 2 – Demographic data and attributed causes of all cases of SD

Case	Gender	Age	Attributed cause of SD	Additional features
**1**	M	24	Atherosclerotic CAD (type 1 MI)	Histological MI, 3-vessel disease with multiple epicardial and intramyocardial coronary thrombosis/embolization
**2**	M	36	Atherosclerotic CAD (type 2)	2-vessel disease (50-75%), LV dilation
**3**	M	40	Atherosclerotic CAD (type 2 MI)	1-vessel disease (>75%), mild LV dilation
**4**	F	34	Atherosclerotic CAD (type 2)	1-vessel disease (50-75%), histological MI as well as myocardial fibrose and scar in the myocardium affected by the affected artery
**5**	M	38	Atherosclerotic CAD (type 2 MI)	3-vessel disease (>75%), LVH, myxomatous mitral valve disease
**6**	M	38	Atherosclerotic CAD (type 1 MI)	Histological MI, 1-vessel disease
**7**	M	37	Atherosclerotic CAD (type 1)	3-vessel disease, LV dilation
**8**	M	34	Atherosclerotic CAD (type 2 MI)	1-vessel disease, LVH
**9**	M	31	Atherosclerotic CAD (type 2 MI)	2-vessel disease (>90% and >75%), LVH, VD and RA dilation
**10**	F	38	Atherosclerotic CAD (type 1 MI)	3-vessel disease, LVH and LV dilation, with interstitial and replacement-type myocardial fibrosis; pulmonary edema (70%)
**11**	F	28	Atherosclerotic CAD (type 2 MI)	3-vessel disease, mild degenerative mitral valve disease; significant heart autolysis
**12**	M	35	Atherosclerotic CAD (type 1 MI)	Histological MI, 1-vessel disease; chronic steatohepatitis
**13**	M	39	Atherosclerotic CAD (type 1 MI)	1-vessel disease, LVH, with interstitial and replacement-type myocardial fibrosis
**14**	M	30	Atherosclerotic CAD (type 1 MI)	2-vessel disease, LVH
**15**	M	30	Atherosclerotic CAD (MI with wall rupture and hemopericardium)	No anatomopathologic report available
**16**	M	39	Atherosclerotic CAD (type 1)	3-vessel disease, LVH
**17**	M	34	Atherosclerotic CAD (type 1 MI)	Histological MI, 1-vessel disease; pulmonary edema (60%)
**18**	M	36	Atherosclerotic CAD (type 1 MI)	1-vessel disease, LVH, LV dilation, interstitial and replacement-type myocardial fibrosis
**19**	M	39	Atherosclerotic CAD (type 2 MI)	Histological MI, 1-vessel disease (>90%)
**20**	M	36	Atherosclerotic CAD (type 2)	1-vessel disease (>75%), LVH
**21**	M	29	Atherosclerotic CAD (type 2 MI)	2-vessel disease (>75%)
**22**	F	40	Atherosclerotic CAD (type 1 MI)	1-vessel disease; pulmonary edema (20%); hepatic steatosis
**23**	M	40	Atherosclerotic CAD (type 1)	1-vessel disease
**24**	M	36	Atherosclerotic CAD (type 2)	1-vessel disease (75-90%), large area of histological MI, as well as multiple myocardial scars
**25**	M	28	Atherosclerotic CAD (type 1 MI)	2-vessel disease, LVH, interstitial myocardial fibrosis and extensive area of replacement-type fibrosis with associated LV thrombus
**26**	M	31	Atherosclerotic CAD (type 2 MI)	3-vessel disease (50-75%), LVH
**27**	F	30	Atherosclerotic CAD (type 1 MI)	3-vessel disease, LVH; pulmonary edema (20%)
**28**	M	30	Atherosclerotic CAD (type 2 MI)	3-vessel disease (>75%), LV dilation
**29**	M	35	Atherosclerotic CAD (type 2 MI)	2-vessel disease (>75%)
**30**	M	36	Atherosclerotic CAD (type 2 MI)	1-vessel disease (50-75%), LV mild dilation
**31**	M	39	Atherosclerotic CAD	Histological MI, LV dilation; coronary arteries not sent for anatomopathological analysis
**32**	M	28	Atherosclerotic CAD (type 1)	2-vessel disease, LVH and evolution towards dilation
**33**	F	37	Atherosclerotic CAD (type 2 MI)	1-vessel disease (50-75%), LVH; adrenal cortical nodular hyperplasia
**34**	M	27	Atherosclerotic CAD (type 2 MI)	2-vessel disease (>75%), LV dilation, mild myxomatous mitral valve disease
**35**	M	32	Atherosclerotic CAD (type 2 MI)	LVH; persistent left superior vena cava draining into the coronary sinus
**36**	M	38	Atherosclerotic CAD (type 2 MI)	3-vessel disease (50-75%), LV dilation
**37**	M	40	Atherosclerotic CAD (type 1 MI)	Histological MI, 3-vessel disease, LVH, with myocardial scar
**38**	M	36	LVH and interstitial fibrosis	Mild RV dilation
**39**	M	24	LVH with interstitial and replacement-type myocardial fibrosis	Extensive anterolateral scar with compensatory hypertrophy, RV dilation, small caliber left coronary arteries
**40**	M	29	LVH	Associated myxomatous mitral valve disease

**41**	M	39	LVH with evolution towards dilation	Diffuse glomerulosclerosis and focal lamellar sclerosis suggestive of diabetic and/or hypertensive nephropathy
**42**	M	37	LVH with evolution towards dilation	RV hypertrophy and dilation
**43**	F	39	LVH and interstitial fibrosis	Non-significant CAD
**44**	F	11	LVH with interstitial and replacement-type myocardial fibrosis	Extensive area of myocardial scar; BALT hyperplasia, probably secondary to previous myocarditis.
**45**	M	34	LVH with evolution towards dilation	
**46**	M	31	LVH with evolution towards dilation	Non-significant CAD, acute pulmonary edema (70%) and generalized vascular congestion; Hypertensive glomerular arteriosclerosis
**47**	M	34	LVH	No anatomopathologic report available
**48**	M	33	LVH	Non-significant CAD, right chamber dilation with posterior RA sacular aneurysm; hepatic steatosis
**49**	F	39	LVH with evolution towards dilation	Hepatic steatosis
**50**	M	29	LVH	Non-significant CAD; heart autolysis and deformation
**51**	M	37	LVH with evolution towards dilation	RV hypertrophy, acute pulmonary edema (25%)
**52**	M	23	LVH with evolution towards dilation	Biventricular hypertrophy; Non-significant CAD; adrenal cortical adenoma
**53**	F	40	LVH with evolution towards dilation	Non-significant CAD
**54**	M	40	LVH	Patent foramen *ovale*
**55**	M	13	HCM	Heart weight: 220g, left ventricular free wall thickness of 13-16mm. Myocyte disarray. Pathogenic MYBPC3 gene mutation.
**56**	M	30	Obstructive HCM	No anatomopathologic report available
**57**	M	14	Obstructive HCM	Heart weight: 740g, interventricular septum thickness of 35mm. Myocyte disarray. Two pathogenic MYBPC3 gene mutations: *antemortem* diagnosis of father-inherited mutation and *postmortem* diagnosis of mother-inherited mutation. Toxicology: atenolol in the therapeutic range.
**58**	M	25	Acute pulmonary embolism	
**59**	M	37	Acute pulmonary embolism	
**60**	F	40	Acute pulmonary embolism	Inferior limb deep venous thrombosis
**61**	F	32	Acute pulmonary embolism	Inferior limb deep venous thrombosis
**62**	F	21	Acute pulmonary embolism	
**63**	F	34	Acute pulmonary embolism	
**64**	F	39	Acute pulmonary embolism	Inferior limb deep venous thrombosis
**65**	F	40	Acute pulmonary embolism	Inferior limb deep venous thrombosis; RV dilation; Bilateral pleural effusion; Acute salpingitis
**66**	F	32	Acute pulmonary embolism	Inferior limb deep venous thrombosis
**67**	F	36	Acute pulmonary embolism	Inferior limb deep venous thrombosis
**68**	M	32	Acute pulmonary embolism	Inferior limb deep venous thrombosis
**69**	F	27	Acute pulmonary embolism	Invasive colic adenocarcinoma (stage T4N3M1)
**70**	F	30	Acute pulmonary embolism	Inferior limb deep venous thrombosis
**71**	F	36	Acute pulmonary embolism	Inferior limb deep venous thrombosis
**72**	M	18	LV dilation - possibly post-myocarditis	Heart weight: 440g. Associated chronic portal plus interface hepatitis, and BALT hyperplasia raising the hypothesis of subacute myocarditis due to a viral infection or auto-immune disease
**73**	F	33	LV dilation	Mild to moderate interstitial fibrosis
**74**	F	37	LV dilation - possible post-partum (3 months) cardiomyopathy	Hepatic steatosis (> 85%), with diminished hepatic reserve
**75**	F	37	LV dilation - possible ethanolic cardiomyopathy	Heart weight: 410g. Steatohepatitis and hepatomegaly; Chronic interstitial nephritis; Prior history of chronic alcoholism
**76**	M	35	LV dilation	
**77**	F	17	LV dilation - possible post-partum (4 months) cardiomyopathy	
**78**	M	36	LV dilation	Heart weight: 170g. Excentric atherosclerostic lesions (~50%)
**79**	M	27	LV dilation	Heart weight: 400g.
**80**	M	36	Ischemic heart disease	Heart weight: 700g. Complicated with acute pulmonary edema (80%)

**81**	M	35	LV dilation - possible ethanolic cardiomyopathy	Pulmonary edema (80-90%); prior history of chronic alcoholism and drug dependance
**82**	F	31	Myxomatous mitral valve disease - mitral valve prolapse	Mild LV hypertrophy and dilation
**83**	M	39	Severe aortic stenosis	No anatomopathological evaluation requested
**84**	M	30	Myxomatous mitral valve disease	Mitral valve leaflets ballooning and myxomatous degeneration; LVH, peri-valvular basal ventricular interstitial fibrosis
**85**	M	36	Myxomatous mitral and tricuspid valve disease	LVH, RV and bi-atrial dilation; patent foramen ovale
**86**	M	39	Severe aortic stenosis	Significant heart autolysis. Aortic valve with degenerative changes and coarse calcifications.
**87**	F	38	Myxomatous mitral valve disease - mitral valve prolapse	
**88**	M	40	Degenerative mitral valve disease	Mitral valve leaflet ballooning and severe thickning, calcification of one tendinous chordae; LVH and LV and LA dilation
**89**	M	23	Acute myocarditis	Acute pericarditis
**90**	M	5	Acute myocarditis	Neutrophilic infiltrate suggesting bacterial etiology; BALT hyperplasia
**91**	M	15	Acute myocarditis	Mild LV dilation and interstitial myocardial fibrosis; mononuclear cells infiltrate suggesting viral etiology
**92**	F	23	Acute myocarditis	Small foci of myocardial scar; myocarditis with associated peri-coronary inflammation with eosinophil-rich mixed cellular tissue infiltrates
**93**	M	36	Acute myocarditis	Myocarditis with associated pneumonia with neutrophilic infiltrates suggestion bacterial etiology
**94**	F	40	Acute Heart Failure	Acute pulmonary edema (85%), generalized vascular congestion, mild RV dilation
**95**	M	38	Acute Heart Failure	Acute pulmonary edema (80%), generalized vascular congestion; Chronic hepatitis
**96**	M	28	Acute Heart Failure	Acute pulmonary edema (80%), generalized vascular congestion; Diffuse microvesicular hepatic steatosis
**97**	F	29	Acute Heart Failure	Acute pulmonary edema (90%), generalized vascular congestion
**98**	M	33	Acute Heart Failure	Acute pulmonary edema (55%)
**99**	M	17	DeBakey type II aortic dissection and pericardial tamponade	Originating in a pre-coarctation aneurysm with chronic peri-aortic inflammation; bicuspid aortic valve; peri-coronary inflammation; biventricular hypertrophy.
**100**	M	32	DeBakey type I aortic dissection and pericardial tamponade	Ascending aorta sacular aneurysm, aortic atherosclerosis and media degeneration with acid mucopolysaccharide deposition; cardiopathy with biventricular hypertrophy; 1-vessel significant disease (50-75%)
**101**	M	36	DeBakey type II aortic dissection and pericardial tamponade	Associated acute pulmonary root dissection; significant heart autolysis
**102**	M	38	DeBakey type I aortic dissection and pericardial tamponade	LV dilation, non-significant CAD
**103**	M	26	DeBakey type II aortic dissection and pericardial tamponade	Aneurysm from the ascending to the thoracic descending aorta, with acute intra-aneurysmal thrombosis; Non-significant CAD; pulmonary edema (80%)
**104**	M	27	Congenital Heart Disease	No anatomopathological evaluation requested
**105**	F	36	Corrected Congenital Heart Disease	Bicuspid pulmonary valve and sub- and supra-valvular stenosis corrected with a pulmonary trunk patch; cardiopathy with biventricular hypertrophy, interstitial and replacement-type fibrosis, tricuspid annulus dilation.
**106**	M	31	Corrected Congenital Heart Disease	Pulmonary valve with dysplastic cuspids and a calcified patch in the RV outflow tract; cardiopathy with biventricular dilation, hypertrophy, interstitial and replacement-type fibrosis involving predominantly the RV; Pulmonary edema (35%)
**107**	M	32	Congenital Heart Disease	LVH and LV dilation, tendinous chordae with anomalous insertion into the interventricular septum conditioning mitral valve leaflet restriction; pulmonary edema (85%)

**108**	M	37	Congenital Heart Disease	Pulmonary artery agenesia, interatrial communication, persistent left superior vena cava draining into aneurysmatic coronary sinus, left atrial and mitral valve hypoplasia
**109**	F	35	Left ventricular interstitial fibrosis	Myocardial scars; Non-significant CAD; Pulmonary edema (40%)
**110**	M	40	Left ventricular interstitial fibrosis	Mild and multifocal interstitial fibrosis; mixed diffuse hepatic steatosis; chronic gastritis
**111**	F	30	ARVC	Severe RV dilation, fat replacement in the RV apex, outflow and inflow tract and focal LV involvement
**112**	M	24	Acute left main coronary artery dissection	Originating from medial tunica degeneration of the ascending aorta; Mitral valve myxomatous thickening and ballooning, thin and elongated tendinous chordae – suggesting a hereditary Marfan Syndrome
**113**	M	21	Acute bronchopneumonia	
**114**	M	20	Acute lobar pneumonia	
**115**	M	20	Acute bronchopneumonia	
**116**	M	24	Acute bronchopneumonia	LV dilation; Portal chronic hepatitis
**117**	M	25	Acute lobar pneumonia	
**118**	M	19	Acute bronchopneumonia	Hepatic macrovesicular steatosis
**119**	M	27	Acute lobar pneumonia	
**120**	M	36	Acute lobar pneumonia	
**121**	M	39	Acute bronchopneumonia	
**122**	M	39	Acute bronchopneumonia	
**123**	M	40	Acute lobar pneumonia	
**124**	M	40	Acute purulent bronchitis	LVH with evolution towards dilation
**125**	M	24	Acute purulent bronchitis	LVH with evolution towards dilation
**126**	M	34	Acute panlobar pneumonia	Kidney abcess
**127**	M	38	Acute bronchopneumonia	LVH, 1-vessel disease (>75%)
**128**	M	37	Acute lobar pneumonia	
**129**	M	33	Acute laringitis	LVH with evolution towards dilation, non-significant CAD; Mixed hepatic cirrhosis with signs of activity
**130**	M	15	Acute severe amygdalitis with proeminent lymphoid follicular hyperplasia	LVH, anomalous tendinous chordae; Splenic follicular hyperplasia
**131**	M	29	Acute alveolar hemorrhage	Non-significant CAD
**132**	M	30	Acute alveolar hemorrhage	
**133**	M	39	Acute alveolar hemorrhage	
**134**	M	30	Acute difuse alveolar disease	
**135**	M	24	Acute difuse alveolar disease	LVH; portal chronic hepatitis
**136**	M	33	Acute difuse alveolar disease	
**137**	M	13	Acute pulmonary edema in the context of Still's Disease	Prior history of idiopathic juvenile systemic arteritis
**138**	M	38	Acute pulmonary thrombosis in the context of sickle cell anemia crisis	Prior history of Sickle Cell Anemia
**139**	M	38	Hemorrhagic stroke	Secondary to spontaneous basilar artery rupture
**140**	F	40	Hemorrhagic stroke	
**141**	M	39	Hemorrhagic stroke	
**142**	M	28	Hemorrhagic stroke	Secondary to aneurysm rupture
**143**	M	23	Generalized tonic-clonic seizure	
**144**	F	37	Generalized tonic-clonic seizure	Prior diagnosis of epilepsy
**145**	M	37	Generalized tonic-clonic seizure	Prior diagnosis of epilepsy
**146**	F	40	Generalized tonic-clonic seizure	Prior diagnosis of Ramsay-Hunt Syndrome
**147**	M	38	Subarachnoid hemorrhage	
**148**	F	39	Subarachnoid hemorrhage	Acute pulmonary edema
**149**	M	30	Acute meningitis	
**150**	F	30	Brain ventriculitis and acute cervical medullary hemorrhage	Colloid cyst in the third cerebral ventricle; Patent foramen ovale; right coronary artery with two ostia; coronary arteries with diminished caliber
**151**	F	35	Acute peritonitis secondary to intestinal perforation	
**152**	M	40	Acute peritonitis secondary to gastric ulcer perforation	Prior history of drug abuse and hepatitis C viral infection

**153**	F	39	Acute peritonitis secondary to gastric ulcer perforation	
**154**	F	26	Gastric hemorrhage	Mild LVH, non-significant CAD
**155**	F	31	Granulomatous necrotizing hepatitis	Mild myxomatous mitral valve disease; Mild vascular congestion
**156**	M	40	Lymphoproliferative disease	
**157**	F	36	Intravascular disseminated coagulation secondary to severe sepsis to Streptococcus suis	Pleural, pericardial and peritoneal hematic effusion; Oral mucosal bleeding
**158**	F	39	Ulcerated infected uterine cervix carcinoma	
**159**	M	35	Severe hypoglycemia in the context of insulin-treated DM	Severe hypoglycemia measures by pre-hospital team; Prior history of severe hypoglycemia episodes

Legend: ARVC – Arrhythmogenic Right Ventricle Cardiomyopathy, BALT – bronchus-associated lymphoid tissue, CAD – coronary artery disease, HCM – Hypertrophic Cardiomyopathy, LA – left atrium, LV – left ventricle, LVH – Left Ventricular Hypertrophy, MI – Myocardial Infarction, MYBPC3 – Myosin Binding Protein C3, RA – right atrium, RV – right ventricle
