# Supplementary material for: Sudden death in young South European population: a cross-sectional study of postmortem cases
Source: Sci Rep. 2023 Dec 20;13:22734. doi: 10.1038/s41598-023-47502-0 (PMC10733430; doi:10.1038/s41598-023-47502-0)
Supplement: Supplementary file 3 — Supplementary Information 3. [file 41598_2023_47502_MOESM3_ESM.pdf]

Supplementary material 3 – Global variation in reporting of the etiology of SCD

Study<br>Location	Population	Age<br>Sex<br>Ethnicity	Main Findings	Young adults
**Braggion-Santos et al. 2015 Ribeirão Preto, Brazil**(41)	Necropsy study<br>2006-2010<br>889 cases of SCD	62.7±13.2<br><br>♀33%<br><br>75% Caucasian<br>11% Black<br>13% Mixed race<br>1% Asian	64% Acute coronary syndrome<br>32% Cardiomyopathy* (ischemic and non-ischemic; 5.5% Chagas disease)<br>0.9% Myocardial bridge<br>0.1% Myocarditis<br>0.1% Coronary artery anomaly<br>2.7% undetermined	<40 years<br>N=43 (4.5%)<br><br>10-19 years: 0.3%<br>20-29 years: 0.7%<br>30-39 years: 3.5%<br><br>No details on etiology
**Bonny et al. 2017 Douala, Cameroon**(35)	Health records review<br>2013<br>autopsies not performed systematically<br>27 cases of SCD	♀35 (IQR 16)<br>♂56 (IQR 42)<br><br>♀48%	Out of hospital cardiac arrest in 63% (n=17)<br>One patient experienced chest pain consistent with ACS before dying<br>4 patients had known heart failure, but no information on LVEF<br>37% were nocturnal deaths, with 11% during sleep	Age 18-29:<br>incidence 11.9 per 100.000<br>Age 30-39:<br>Incidence 42 per 100.000<br>No details on etiology
**Ndoye et al. 2019 Dakar, Senegal**(42)	Necropsy study<br>2000-2006<br>235 cases of SCD	46.9 (range 17-86)<br><br>♀22.5%<br><br>96.1% Black<br>3.9% Caucasian	54.9% Cardiomyopathies (49.4% DCM & 5.5% HCM)<br>35.3% MI<br>3.4% Valvulopathies<br>2.5% Aortic dissection<br>0.9% Pericarditis<br>0.4% Atrial septal defect	20-39 years n=47<br>17 due to MI<br>Cardiomyopathies were more frequent in ♀20-29 and ♂50-59
**Darre et al. 2021 Lomé, Togo**(43)	Necropsy study<br>2009-2018<br>173 cases of SCD	43±0.3<br><br>♀36.5%	59.0% MI<br>19.1% Coronary thrombosis without evidence of MI<br>13.3% Chronic IHD<br>6.4% Rupture of aortic aneurysm<br>1.7% Endocarditis<br>0.6% Pericarditis	No details on etiology, but average age was 43
**Rotimi et al. 1998 Osun, Nigeria**(44)	Necropsy study<br>1991-1995<br>50 cases of SCD	Age range 28-80<br><br>♀30%	68% hypertensive heart disease with acute LV failure<br>14% hemorrhagic stroke<br>6% DCM<br>4% HCM<br>4% congestive HF<br>4% MI	21-40 years<br>n=8<br><br>No details on etiology
**Allouche et al. 2013 Tunisia**(45)	Necropy study<br><br>32 cases of sudden death during sports	Age 33.2<br>(range 25-79)<br><br>♀15.6%	69% died during sports activity<br><br>96.9% cardiac cause (n=31)<br><br>>35 years (9 cases)<br>77.8% MI<br>22.2% HCM	<35 years<br>(n=22)<br>40.9% HCM<br>13.6% ARVC<br>9% Myocardial bridge<br>9%MI<br>4.5% Aortic stenosis<br>4.5% Congenital anomalies of the coronary arteries<br>4.5% Aortic dissection<br>4.5% DCM<br>18.2% Blank autopsy
**Aizaki et al. 1997 Kitasato, Japan**(46)	Necropsy study<br>1994-1996<br>72 cases of SCD	47.5 years<br>(range 20-60)<br><br>♀20.3%	69.4% CAD<br>4.2% Anomalies of coronary arteries<br>9.7% Dissecting aneurysms<br>8.3% Pokkuri disease (SUNDS)<br>8.3% Other cardiac diseases	20-39 years<br>CAD or anomaly of the coronary arteries 50% to 2/3 of SCD<br><br>Pokkuri disease (SUNDS)<br>1/3 to 1/6

**Ikeda et al. 2007 Hisayama-cho, Japan**(47)	Epidemiology study on causes of SCD	N.A.	30-35% cardiomyopathies: HCM & DCM<br>50-60% acute or chronic CAD<br>10% inherited arrhythmias	N.A.
**Hua et al. 2009 Yuxian, Kelamayi, Beijing & Guangzhou, China**(48)	Epidemiology study<br>Autopsy rarely performed<br>12 months 2005-06<br>284 cases of SCD	66.7±16.9<br><br>♀45.8%	Study focused on incidence rates according to age, and presence of previously known cardiovascular risk factors or conditions	No details on etiology
**Bagnall et al. 2016 Australia & New Zealand**(28)	Nationwide Prospective collection of data 2010-2012<br>Necropsy<br>490 cases of SCD	1-35 years of age<br><br>♀28%	24% CAD<br>16% Cardiomyopathies (DCM, HCM, ARVC)<br>7% Myocarditis<br>4% Aortic Dissection<br>40% Unexplained SCD<br><br>A clinically relevant cardiac gene mutation was identified in 27% of unexplained SCD cases	SCD aged 1-35
**Eckart et al. 2011 USA**(49)	Military Personnel<br>1998-2008<br>902 cases of SCD	38±11<br><br>♀45.8%<br><br>67.4% Caucasian<br>24.1% African American<br>2.1% Pacific Islander<br>1.9% Asian	≥35 years of age<br>N=604<br>73.2% Atherosclerotic disease<br>10.6% Sudden unexplained death<br>3.5% Idiopathic DCM<br>3.1% HCM<br>2.5% Hypertensive cardiomyopathy<br>2.2% Myocarditis<br>2.1% Ischemic cardiomyopathy<br>1.5% Other<br>1.0% ARVC<br>0.2% Anomalous coronary artery	<35 years of age<br>N=298<br>41.3% Sudden unexplained death<br>23.2% Atherosclerotic disease<br>12.8% HCM<br>5.7% Myocarditis<br>4.7% Idiopathic DCM<br>4.0% Anomalous coronary artery<br>3.7% Hypertensive cardiomyopathy<br>2.7% Other<br>1.3% ARVC<br>0.7% Ischemic cardiomyopathy
**Chugh et al. 2004 Oregon USA**(50)	Prospective study with circumstance of death, medical records and available autopsy data<br><br>12 months 2002-03<br><br>353 cases of SCD (41 with autopsy)	Median age: 69 years<br><br>♀45.8%	≥35 years<br>N=29<br>76% CAD<br>7% Congenital heart disease<br>4% Cardiac rupture<br>3% Myocarditis<br>3% Aortic dissection	<35 years<br>N=12<br><br>No changes were identified
**Maron et al. 2009 USA**(51)	Nationwide Competitive athletes<br>1980-2006<br><br>1866 cases of Sudden Death (or survivors)<br>1236 with autopsy	19±6<br><br>♀11%<br><br>55% White<br>36% Black<br>3% Hispanics<br>1.2% Asians<br>0.2% Native Americans	1049 cardiovascular cause<br>36% HCM<br>17% Coronary artery anomalies<br>6% Myocarditis<br>4% ARVC<br>4% Channelopathies (23 LQTS and 2 BrS)<br>3% Commotio cordis<br>2.4% Mitral valve prolapse<br>2.4% Myocardial bridge<br>2.2% CAD<br>2.2% Aortic rupture	Range 8 to 39 years

			1.8% Aortic stenosis<br>1.6% DCM<br>1.3% Wolff-Parkinson White syndrome<br>1.0% Other	
**de Noronha et al. 2009 London UK**(52)	Necropsy study<br>Young Athletes<br>1996-2008<br>118 cases of SCD	27.9±12.5<br><br>♀4%<br><br>96% White<br>4% Afro-Caribbean	>35 years (max 59)<br>N=29<br>82.8% Cardiomyopathy<br>3.4% Normal heart<br>13.8% Coronary artery pathology<br>0% Other	≤ 35 years<br>N=89<br>55.1% Cardiomyopathy<br>29.2% Normal heart<br>7.9% Coronary artery pathology<br>7.9% Other

Legend: BrS – Brugada syndrome, CAD – coronary artery disease, DCM – dilated cardiomyopathy, HCM – hypertrophic cardiomyopathy, HF – heart failure, IHD – ischemic heart disease, IQR – interquartile range, LQTS – Long QT syndrome, MI – myocardial infarction, SCD – sudden cardiac death, SUNDS - sudden unexplained nocturnal death syndrome
